# TIM-3/Gal-9 interaction induces IFNγ-dependent IDO1 expression in acute myeloid leukemia blast cells

**DOI:** 10.1186/s13045-015-0134-4

**Published:** 2015-04-16

**Authors:** Valentina Folgiero, Loredana Cifaldi, Giuseppina Li Pira, Bianca Maria Goffredo, Luciana Vinti, Franco Locatelli

**Affiliations:** Department of Pediatric Hematology and Oncology, IRCCS Bambino Gesù Children’s Hospital, Viale di San Paolo 15, 00146 Rome, Italy; Department of Laboratory Medicine, IRCCS Bambino Gesù Children’s Hospital, Rome, Italy; Department of Pediatric Science, University of Pavia, Pavia, Italy

**Keywords:** AML, IDO1, Immune escape, NK cells, Galectin-9

## Abstract

NK cells expressing TIM-3 show a marked increase in IFNγ production in response to acute myeloid leukemia (AML) blast cells that endogenously express Gal-9. Herein, we demonstrate that NK cell-mediated production of IFNγ, induced by TIM-3/Gal-9 interaction and released in bone marrow microenvironment, is responsible for IDO1 expression in AML blasts. IDO1-expressing AML blasts consequently down-regulate NK cell degranulation activity, by sustaining leukemia immune escape. Furthermore, the blocking of TIM-3/Gal-9 interaction strongly down-regulates IFNγ-dependent IDO1 activity. Thus, the inhibition of TIM-3/Gal-9 immune check point, which affects NK cell-dependent IFNγ production and the consequent IDO1 activation, could usefully integrate current chemotherapeutic approaches.

**Electronic supplementary material:**

The online version of this article (doi:10.1186/s13045-015-0134-4) contains supplementary material, which is available to authorized users.

## Findings

The interaction between T-cell immunoglobulin mucin (TIM)-3 and Galectin-9 (Gal-9) mediates signaling pathways involved in infection, autoimmunity, inflammation, peripheral tolerance, and tumor immunity [1]. TIM-3 is a type I membrane glycoprotein, highly expressed on murine and human natural killer (NK) cells [2]. Gal-9 is a S-type β-galactoside-binding lectin, known as a ligand for TIM-3, and highly expressed in tissues of immune system such as lymph nodes, thymus, spleen, and bone marrow [3]. Recent analyses revealed acute myeloid leukemia (AML) blast cells to be positive for Gal-9 expression [4].

NK-cell function may play a role in antitumor surveillance that is distinct from MHC-restricted cytolytic activity of T cells [5]. Recent studies suggest that NK cell-based immunotherapy may be an effective approach for patients with leukemia, and emerging strategies based on adoptive transfer of NK cells are now under investigation [6-8]. Indoleamine 2,3-dioxygenase 1 (IDO1), an enzyme able to degrade tryptophan into kynurenine, is a nodal mediator of pathogenic inflammation and immune escape in cancer. IDO1 activation results into the functional suppression of T and NK cells and the generation of T regulatory cells (Treg). The gene encoding IDO1 was the first interferon (IFN)-activated gene to be described [9].

In our recent paper by Folgiero et al., we demonstrated that in 51% of samples obtained from AML pediatric patients, IDO1 was up-regulated in response to IFNγ and negatively correlated with prognosis. However, until now, the microenvironmental source of IFNγ in childhood AML remained to be identified [10]. Since NK cells are the main cell population expressing TIM-3 in response to cytokine stimulation and as the engagement of TIM-3 with Gal-9 ligand induces significant increase in IFNγ production by NK cells [4], we speculated that TIM-3/Gal-9 interaction in BM could be responsible of IDO1 induction in AML blasts and could contribute to mediate the consequent immune escape mechanism.

**Figure 1:**
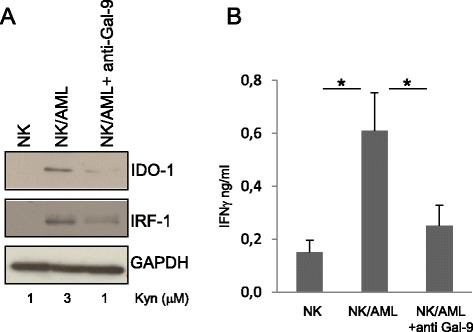
NK/AML co-culture induces TIM-3/Gal-9-dependent IDO-1 activation. BM samples from children with AML at diagnosis were processed by Ficoll-Paque Plus to obtain BM mononuclear cells. PBMC cells processed from buffy coat preparations of healthy donors were cultured for 10 days on a feeder layer of irradiated RPMI 8866 cells to obtain polyclonal expansion of NK cells. **(A)** IDO1 and IRF1 expression on protein extracts from 24-h culture (5:1 ratio) of AML blasts and AML blasts pre-treated for 1 h with anti-Gal-9 antibody with NK cells compared with NK cells alone. GAPDH Ab was used as loading control. Supernatants were collected and used for the measurement of kynurenine levels by RP-HPLC in the different culture conditions. Results shown are representative of 3 AML samples. **(B)** ELISA assay for IFNγ release was performed in the supernatants of experimental co-cultures. Bars are representative of mean values and standard deviation recorded in three independent experiments performed in duplicate (*P < 0. 05).

**Figure 2:**
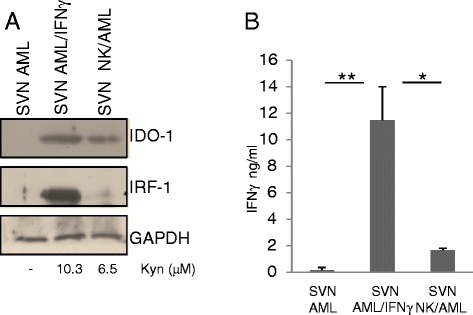
NK/AML co-culture supernatants induce IFNγ-dependent IDO1 activation. Supernatants obtained from AML alone, stimulated overnight with rh-IFNγ 100 ng/ml and co-cultured with NK cells were used to culture AML blasts that do not spontaneously express IDO1. **(A)** IDO1 and IRF1 expression was evaluated by western blotting and compared with kynurenine release in the new culture supernatants. GAPDH Ab was used as loading control. Results shown in the figure are representative of three AML samples. **(B)** ELISA assay for IFNγ quantification was performed in the new three culture conditions. Bars are representative of mean values and standard deviation recorded in three independent experiments performed in duplicate (*P < 0.05; **P < 0.01).

We evaluated the level of Gal-9 expression in AML blasts and confirmed that NK cells obtained from peripheral blood of healthy donors were TIM-3 positive (Additional file 1: Figure S1A, B). To mimic leukemia-conditioned microenvironment, NK cells from healthy donors were co-cultured with AML blasts that did not spontaneously express IDO1. After 24 h of co-culture, NK/AML cells were assayed for IDO1 expression by western blotting. As shown in Figure 1A, co-culture of NK cells with AML blasts resulted positive for IDO1 expression when compared with NK cells cultured in the absence of leukemia blasts. NK/AML co-culture performed in the presence of inhibitory anti-Gal-9 antibody (Ab) revealed a strong down-regulation of IDO1 induction. The activity of IDO1 in the different culture conditions was validated by kynurenine production resulting from its enzymatic activity. We also quantified IFNγ production in the supernatants of co-cultures by ELISA assay. As shown in Figure 1B, NK/AML co-culture induces a fivefold increase in IFNγ production compared to culture of NK cells alone. The addition of anti-Gal-9 Ab to cultures resulted in a significant reduction of IFNγ produced by NK cells. To monitor the effect of IFNγ production, we evaluated the level of interferon regulatory factor 1 (IRF1), a transcription activator of genes induced by interferon. As shown in Figure 1A, IRF1 shows the same trend of expression of IDO1 and correlates with the amount of IFNγ produced. To emphasize that IDO1 expression in AML blasts depends on NK cell-mediated IFNγ production, we directly stimulated non-IDO1-expressing AML blasts with the supernatant (SVN) obtained from co-cultured NK/AML. As shown in Figure 2A, the SVN derived from the co-culture is able to induce a strong expression of both IDO1 and IRF1 that correlated with the increased enzymatic activity. IDO1 induction was almost comparable with that obtained after addition to culture of recombinant human IFNγ (100 ng/ml). Expression of IDO1 and IRF1 strongly correlated with the level of IFNγ production in all the three culture conditions (Figure 2B). Consistent with previous data [11], we showed that IDO1^+^-AML blasts are able to strongly down-regulate NK-cell degranulation compared to unstimulated AML blasts (Additional file 1: Figure S2). These results demonstrate that in the microenvironment of AML, the dysfunctional effect exerted on NK cells could be due to the AML-IDO1 induced by NK/AML interaction. In particular, IFNγ production mediated by TIM-3/Gal-9 interaction and the consequent IDO1 expression induced in AML blasts could affect NK cell degranulation activity favoring AML immune escape. Furthermore, the ability of anti-Gal-9 Ab to reduce IFNγ production by blocking TIM-3/Gal-9 interaction supports the hypothesis that *in vivo* administration of monoclonal antibodies (mAbs) may successfully integrate current chemotherapeutic approaches, increasing their efficacy. Indeed, recently, administration of mAbs interfering with immune checkpoints, including TIM-3/Gal-9, shows encouraging clinical results [12]. In conclusion, our findings could constitute a definitive proof of the relation, occurring in leukemia microenvironment, between IDO1 induction on AML blasts mediated by NK cell-produced IFNγ and the consequent functional deregulation of NK cells that favors AML immune escape.

## Materials and methods

### Sample collection

Bone marrow samples from patients at the onset of AML were aseptically withdrawn and collected in EDTA-containing tubes. The samples were used to isolate BMMC by density gradient centrifugation by Ficoll-Paque Plus. The cells were either used fresh or were stored in FCS with 10% dimethyl sulfoxide in the vapor phase of liquid nitrogen until the day of experimental manipulation.

### Abs and reagents

PE-conjugated anti-CD56, APC-conjugated anti-CD3, and FITC-conjugated anti-CD107a were purchased from BD Biosciences (Mountain View, CA). APC-conjugated anti-TIM-3 was from R & D System. PE-conjugated anti-Galectin-9 was from Biolegend. Rabbit anti-human IDO (H-101) and anti-IRF-1 (C-20) were purchased from Santa Cruz Biotechnology. Rabbit anti-human GAPDH (D16H11) antibodies were from Cell signaling (Milan, Italy). Horseradish peroxidase (HRP)-conjugated anti-Rabbit was purchased from BioRad (Hercules, CA). Recombinant Human IFNγ was from R & D Systems (Minneapolis, MN).

### NK-dependent IDO-1 induction

PBMC cells obtained from buffy coat preparations of healthy donors were cultured for 10 days on a feeder layer of RPMI 8866 cells, irradiated at 3,000 Gy. After the validation of TIM-3 positivity, NK cells were cultured for 24 h with AML blasts previously validated for Galectin-9 positivity. At the end of NK/AML cell co-culture, the supernatant was in part analyzed to validate IDO-1 activity by HPLC, in part analyzed to quantify IFNγ production and in part used to stimulate a new aliquot of AML blasts for 48 h. The cellular compartment was analyzed by WB for IDO-1 expression. AML cells stimulated or not with IFNγ 100 ng/ml were used as controls.

### Western blotting

Cell pellets were lysed with RIPA buffer [150 mM NaCl, 1% NP-40, 0.5% sodium deoxycholate, 0.1% SDS, 50 mM Tris–HCl (pH = 8), 1 mM PMSF, 1 mM EGTA, 50 mM NaF, 50 mM Na3VO4, and protease inhibitors (Roche, Milan, Italy)]. Cell lysates were incubated on ice for 20 min and clarified by centrifugation at 14,000 rpm for 20 min. Cell extracts obtained with RIPA buffer were boiled for 5 min at 95°C and analyzed by 10% SDS-PAGE. Samples were transferred onto nitrocellulose membrane (Bio-Rad, Milan, Italy). Blots were probed with primary antibodies, washed, and developed with HRP-conjugated rabbit or mouse secondary antibodies (Bio-Rad), as appropriate. The bands were quantified densitometrically using the ImageJ software (National Institutes of Health, Bethesda, MD).

### IDO1 activity

Tryptophan and kynurenine levels were measured with reverse-phase HPLC Agilent Technologies 1200. Briefly, sample aliquots (200 μL) were diluted with 200 μL potassium phosphate buffer (0.05 mol/L pH 6.0) containing the internal standard 3-nitro-L-tyrosine (100 μmol/L). Protein was precipitated with 50 μL of trichloroacetic acid (2 mol/L). The capped tubes with the precipitate were immediately vortex-mixed and centrifuged for 10 min at 13,000 *g*. One hundred fifty microliters of the supernatants was transferred into microvials and placed into the autosampling device. The samples were analyzed using a Protocol C18HPH 150 × 4.6 mm 5 μ column (SGE Analytical Science) and a double-pump HPLC apparatus Agilent Technologies equipped with spectrophotometric and fluorescence detectors. Tryptophan was detected by a fluorescence detector at an excitation wavelength of 285 nm and an emission wavelength of 365 nm. Kynurenine and nitrotyrosine were detected by recording UV absorbance at a wavelength of 360 nm. The elution solvent was as follows: buffer A: potassium phosphate solution (0.015 mol/L, pH 6.4) containing 27 mL acetonitrile and buffer B: acetonitrile. Analyses were carried out at a flow rate 1 mL/min a temperature of 25°C in 12 min. The concentration of components was calculated according to peak heights and was compared with both 3-nitro-L-tyrosine as internal standard and with reference curves constructed with L-tryptophan (concentration 10, 20, 30 μmoli/L) and KYN (10, 20, 30 μmoli/L). The intra-daily coefficient of variation was 1.20% for tryptophan and 1.25% for kynurenine. Inter-days coefficient was 3.5% for tryptophan and 3.8% for kynurenine.

### ELISA assay

IFNγ was quantitated using a conventional ELISA assay. Reagents were purchased from Mabtech, Stockholm, Sweden, and used as recommended. Briefly, 96-well plates were coated with the first antibody (1 μg/ml in PBS, 100 μl/well) for 4–12 h at room temperature. After washing twice with PBS, the wells were saturated with PBS/BSA 0.5% for 1 h. After washing, the wells received 100 μl of each sample (either neat or diluted in PBS/BSA 0.5% Tween20 0.01%). Following incubation for 1 h, the wells were washed three times with PBS-Tween. The second biotinylated antibody diluted at 1 μg/ml in PBS/BSA-Tween was added. One hour later, the wells were washed three times with PBS-Tween and alkaline phosphatase-conjugated streptavidin was added at 1 μg/ml in PBS/BSA-Tween. After 1 h, the wells washed three times and the enzyme substrate PNPP (Sigma Aldrich, St. Louis, MO) was added at 1 mg/ml in diethanolamine buffer pH 9. Following a 30–60-min incubation at room temperature, the plates were scanned at 415 nm. IFNγ concentration in the samples was determinate using a titration curve with a cytokine standard by the manufacturer.

### CD107a degranulation assay

NK cells were selected (NK isolation Kit) from buffy coat preparations of consenting blood donors and were processed following the manufacturer’s protocol. NK cells were activated with 50 IU/ml IL-2 for 24 h. After washings with PBS, NK cells were co-cultured for 3 h with either AML blasts and IFNγ-stimulated AML blasts at 1:1, 1:3, and 1:10 effector-to-target (E:T) ratio. K562 (NK-sensitive) or Raji cells (NK-resistant) were used as NK activity control, as previously published. Thereafter, the cells were labeled with PE-conjugated anti-CD56, APC-conjugated anti-CD-3, and FITC-conjugated anti-CD107a antibodies for 20 min at 4°C, followed by flow cytometry analysis. Isotype-matched antibodies from the same manufacturer were used to assess background fluorescence.

## Additional file

Additional file 1: Figure S1. Characterization of TIM-3 and Gal-9 expression. (A) AML blasts obtained from BM samples of AML children were analyzed for Gal-9 expression by FACS analysis. (B) NK cells obtained from healthy donor buffy coats were tested for TIM-3 expression by FACS analysis. Results shown in the figure are representative of three different experiments. **Figure S2.** IDO1-expressing-AML affects NK degranulation activity. NK cells were cultured with AML blasts and with AML blasts pre-treated with IFNγ (1:1 ratio). After 3 h, the cells were harvested and labeled with CD3, CD56, and CD107a antibodies to evaluate NK cells degranulation activity by FACS analysis. Results shown in the figure are representative of three different experiments.
