# Supplementary material for: TIM-3/Gal-9 interaction induces IFNγ-dependent IDO1 expression in acute myeloid leukemia blast cells
Source: J Hematol Oncol. 2015 Apr 16;8:36. doi: 10.1186/s13045-015-0134-4 (PMC4404691; doi:10.1186/s13045-015-0134-4)
Supplement: Additional file 1: Figure S1. — Characterization of TIM-3 and Gal-9 expression. (A) AML blasts obtained from BM samples of AML children were analyzed for Gal-9 expression by FACS analysis. (B) NK cells obtained from healthy donor buffy coats were tested for TIM-3 expression by FACS analysis. Results shown in the figure are representative of three different experiments. Figure S2. IDO1-expressing-AML affects NK degranulation activity. NK cells were cultured with AML blasts and with AML blasts pre-treated with IFNγ (1:1 ratio). After 3 h, the cells were harvested and labeled with CD3, CD56, and CD107a antibodies to evaluate NK cells degranulation activity by FACS analysis. Results shown in the figure are representative of three different experiments. [file 13045_2015_134_MOESM1_ESM.pdf]

Supplementary Figure 1 Folgiero V et al

A

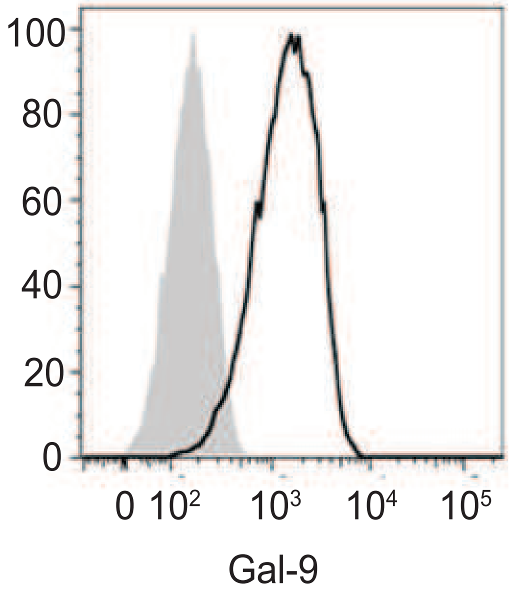

B

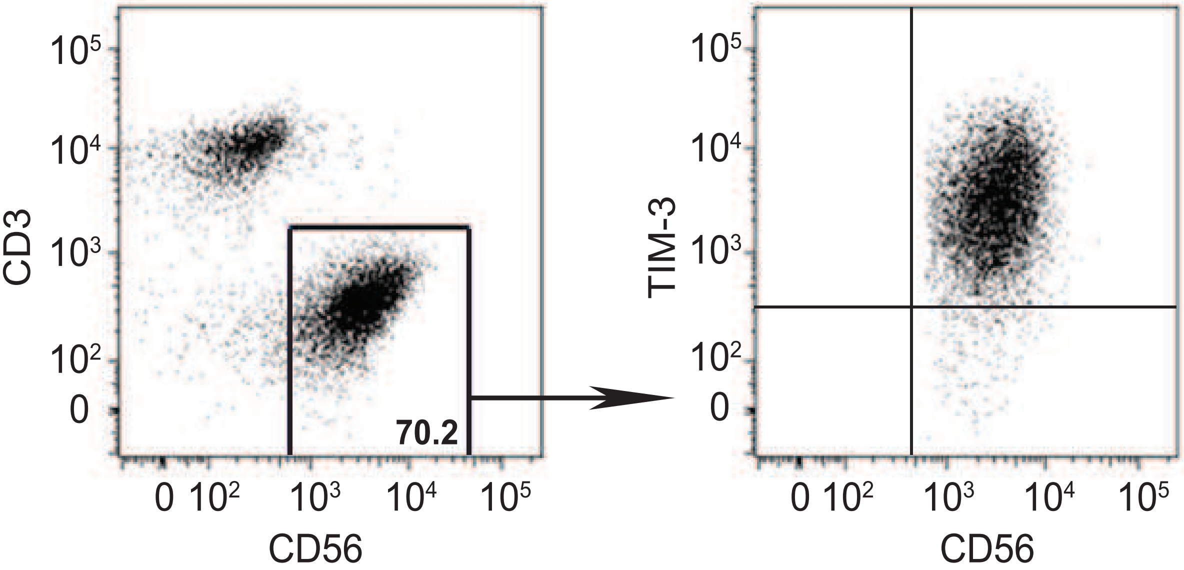

Supplementary Figure 2

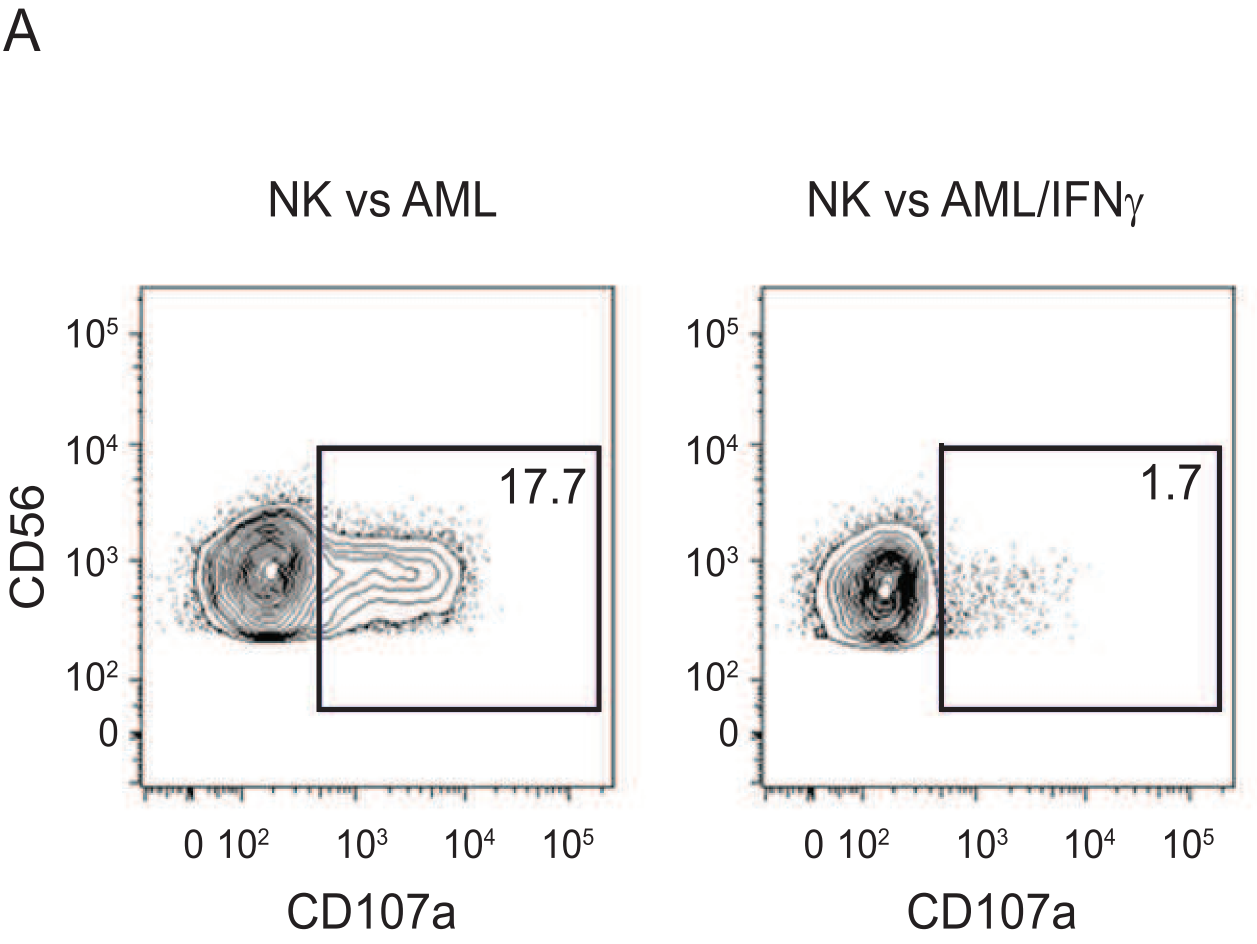

Supplementary Figure Legends Folgiero V et al

Supplementary Figure 1: Characterization of TIM-3 and Gal-9 expression. (A) AML blasts obtained from BM samples of AML children were analyzed for Gal-9 expression by FACS analysis. (B) NK cells obtained from healthy donors buffy coats were tested for TIM-3 expression by FACS analysis. Results shown in the figure are representative of three different experiments.

Supplementary Figure 2: IDO1-expressing-AML affects NK degranulation activity. NK cells were cultured with AML blasts and with AML blasts pre-treated with IFNγ (1:1 ratio). After 3 hours, the cells were harvested and labeled with CD3, CD56 and CD107a antibodies to evaluate NK cells degranulation activity by FACS analysis. Results shown in the figure are representative of three different experiments.
